# Mandibular Advancement Devices Prevent the Adverse Cardiac Effects of Obstructive Sleep Apnea-Hypopnea Syndrome (OSAHS)

**DOI:** 10.1038/s41598-020-60034-1

**Published:** 2020-02-25

**Authors:** Chunyan Liu, Wenjing Kang, Shilong Zhang, Xing Qiao, Xiuchun Yang, Zheng Zhou, Haiyan Lu

**Affiliations:** 1 Department of Orthodontics, School and Hospital of Stomatology, Hebei Medical University & Hebei Key Laboratory of Stomatology, Shijiazhuang, 050017 P.R. China; 2 Department of Periodontology and Dental Hygiene, School of Dentistry, University of Detroit Mercy, Detroit, MI USA; 3 Department of Cardiology, The Second Hospital of Hebei Medical University; Hebei Province, Shijiazhuang, China

**Keywords:** Transcriptional regulatory elements, Respiration, Cryoelectron microscopy

## Abstract

Although considerable research highlights the interactions between obstructive sleep apnea-hypopnea syndrome (OSAHS) and cardiovascular diseases, the effect of mandibular advancement device (MAD) treatment on cardiovascular complications in OSAHS patients remains unclear. We evaluated the effect of OSAHS treatment with MADs on the myocardium. All methods in this study were in accordance with relevant guidelines and regulations of the medical ethics committee in Hospital of Stomatology, Hebei Medical University approved the work. Thirty New Zealand rabbits were randomized into three groups: the control group, Group OSAHS, and Group MAD. Hydrophilic polyacrylamide gel was injected into the soft palate of the rabbits to induce OSAHS. In Group MAD, a MAD was positioned after OSAHS induction. All animals were induced to sleep in a supine position for 4–6 h/day for 8 weeks. Echocardiography was used to determine the structure and function of the heart. The histological changes were detected by optical microscopy and transmission electron microscopy (TEM). The levels of ET-1(endothelin-1) and Ang II (Angiotensin II) in the plasma were measured by an enzyme-linked immunosorbent assay (ELISA). The expression of ET-1 mRNA in heart tissue was detected by RT-PCR. Histological abnormalities, left ventricular hypertrophy, and left ventricular dysfunctions were demonstrated in Group OSAHS, and the abnormities were rescued with MAD treatment. Higher levels of plasma ET-1 and Ang II and elevated expression of ET-1 mRNA in cardiac tissue were detected in Group OSAHS compared with Group MAD and the control group. The blood oxygen saturation was negatively correlated with the levels of ET-1 and Ang II. OSAHS-induced elevated levels of ET-1 and Ang II may be attributed to myocardial structural abnormalities and dysfunction. Early treatment of MADs may play an important role in preventing myocardial damage in OSAHS rabbit model.

## Introduction

Obstructive sleep apnea-hypoxia syndrome (OSAHS) affects 9–38% of the general adult population, 13–33% of men and 6–19% of women, and prevalence rates increase with age^1^. It is characterized by recurrent partial or complete closure of the upper airway during sleep, which leads to oxygen desaturation and arousal from sleep. OSAHS is a potential major risk factor for the morbidity and mortality of many cardiovascular complications^2^, such as hypertension, arrhythmias, myocardial infarction, heart failure, ventricular hypertrophy, systolic and diastolic dysfunctions, as well as stroke^3,4^. OSAHS is associated with a significantly higher risk of hypertension and is regarded as an independent risk factor for hypertension^5^. Evidence indicated that the cardiac dysfunction and cardiomyocyte injury was detected in OSAHS patients and animal modes^6,7^. The cardiac dysfunction might be associated with a decreased coronary tone, and an intact coronary endothelium during OSAHS^8^. Previous studies suggested that OSAHS was contributed to microvascular endothelial dysfunction and augmented vasoconstriction^9^, which was associated with endothelial system. The renin-angiotensin system plays critical roles in maintaining normal cardiovascular functions and regulates extracellular fluid volume and blood pressure. This system may be potentiated by increased sympathetic system activity, and increased levels of insulin and leptin that are frequently present in OSAHS. Sympathetic nervous system overactivity could result in the overproduction of ET-1(endothelin-1) and Ang II (Angiotensin II), which are important to vascular tone and blood pressure. ET-1 and Ang II are mainly produced by endothelium and play an important role in cardiovascular alterations. Ang II induces several forms of cardiac dysfunction including hypertrophy, arrhythmia, and ventricle function failure^10,11^. Sleep apnea patients have been shown to have higher angiotensin II concentrations compared to healthy subjects^12,13^. Therefore, we speculated that OSAHS induced cardiac dysfunction and abnormal structure, which might be associated with ET-1 and Ang II levels.

OSAHS therapies include behavioral treatment^14^, continuous positive airway pressure (CPAP) treatment^15,16^, mandibular advancement devices (MADs)^17,18^, and surgeries to enlarge the upper airway, such as uvulopalatopharyngoplasty (UPPP) and maxilla-mandibular advancement osteotomy^19^. It is popular to use MADs for most OSAHS patients, especially those unable to tolerate CPAP therapy, due to their many advantages, such as being traumatic, convenient, and low cost. It has been shown that OSAHS treatment might reduce the risk of cardiovascular complications. CPAP treatment has been proven to reduce blood pressure^5,20^ and correct left ventricular hypertrophy and systolic and diastolic dysfunctions caused by OSAHS^21,22^. There were limited data on the effects of oral appliance treatment on cardiac function. Both Hoekema *et al*.^23^ and Barnes *et al*.^24^ used echocardiography to image left ventricular structures and function and did not find any effect of oral appliance on this heart function parameter after 3 month of treatment. A recent meta-analysis showed the other studies on MADs treatment on the cardiovascular system associated with OSAHS were limited to the changes in blood pressure, heart rate and fatal cardiovascular events^25^. Therefore, there is a lack of good quality data assessing the effect of MADs on cardiovascular system.

In the present study, we aimed to investigate the changes of cardiac structure and function in the rabbit OSAHS model developed in our laboratory^26,27^ and to determine the effects of OSAHS on cardiac structure and function and the role of MADs in the remedies of possible injuries. This will provide evidence for the clinical treatment of OSAHS with MADs and further explore the possible mechanism of these changes.

## Materials and Methods

All methods in this study were performed in accordance with the medical ethics committee in Hospital of Stomatology, Hebei Medical University. Animal use and care was in accordance with the guidelines of the medical ethics committee for the housing and care of animals bred, supplied and used for scientific purposes. All experiments were performed in accordance with relevant guidelines and regulations. The medical ethics committee in Hospital of Stomatology, Hebei Medical University approved the work (Certificate No. [2017]013).

### Experimental animals

Thirty 6-month-old male New Zealand white rabbits (body weight 3.0–3.5 kg) were randomly divided into three groups, the control group, Group OSAHS, and Group MAD, with 10 animals in each group. The OSAHS rabbits were induced as previously described in our study^26^. In brief, the animals of Group OSAHS and Group MAD were induced to develop OSAHS by gel injection into the center of soft palate, approximately 1.5 cm away from the junction of the hard and soft palates. The MADs were given to the OSAHS rabbits in Group MAD on day 3 after OSAHS development. Animals in the control group were not given any treatment.

### CBCT and polysomnography (PSG)

The animals of all three groups were fixed in a homemade device to simulate the supine position. A CBCT scan of the upper airway was performed using a CBCT machine. The scan parameters were set to 120 KV of voltage, 5 mA of current, 0.3 mm of layer thickness, and 17.8 s of scan time. A single 360° rotation scan was performed from the cranium to the clavicle. Then, the retropalatal space was examined by measuring the sagittal diameter of the upper airway from the top to the 1/4, 1/2, and 3/4 level of the soft palate.

PSG (Rembrandt Embla Polysomnography System, Reykjavik, Iceland) was used to monitor the sleep parameters. PSG recordings were conducted as described in our previous studies^26,27^. Hypopnea was defined as a reduction between 20% and 50% in nasal flow for at least two breaths. Apnea was defined as an absence of airflow at the nose and mouth for longer than two breaths^28^. The apnea-hypopnea index (AHI), the number of apneas and hypopneas per hour during sleep throughout the whole night, was scored for all animals.

### MAD fabrication

The fabrication of MADs was performed according to the previous protocol developed in our laboratory^26^. Briefly, MADs were made of self-curing composite resin with a 30° inclined plane to the long axis of the upper incisors and was bonded to the upper incisors with glass ionomers. The mandible was guided forward approximately 3–4 mm. CT and PSG were used to evaluate the effectiveness of MAD treatment following the protocols described above.

### Morphological changes in the heart

All animals were induced to sleep in a supine position for 4–6 hours per day for eight consecutive weeks. After eight weeks, heart tissues were harvested and embedded in paraffin after fixation in 10% buffered formalin. Sections were stained with hematoxylin/eosin and examined under light microscopy (Olympus, Japan). A portion of the cardiac apex was quickly dissected and fixed in 4% cryopreservation glutaraldehyde solution and then viewed by transmission electron microscopy.

### Structural and functional changes in cardiac tissue examined by echocardiography

Echocardiographic analysis was performed by a high-resolution ultrasound imaging system (Philips Medical Systems, Andover, Massachusetts, USA) with an IE33 transducer. First, the rabbits were held in the supine position, the anterior chest wall was shaved, warm ultrasound gel applied to the chest, and the transducer probe was placed over the left hemithorax. Multiple short axis M-mode images of the left ventricle were obtained, and these images were analyzed for left ventricular (LV) functional parameters in triplicate. The other indices measured included the LV wall thicknesses [LV posterior wall (LVPW) and interventricular septum (IVS)], right ventricle diameter (RVD), and LV chamber dimensions (LVID) as recommended by the American Society of Echocardiography. M-mode recordings detected the left ventricular end-diastolic diameter (LVEDd) and left ventricular endsystolic diameter (LVEDs). The left ventricular endsystolic volume LVESV = 7/(2.4 + LVEDs) × LVEDs3 × 1000, left ventricular end-diastolic volume LVEDV = 7/(2.4 + LVEDd) × LVEDd3 × 1000, and left ventricular ejection fraction (LVEF) = (LVEDV − LVESV)/LVEDV × 100%. Four-chamber echocardiography showed the maximum flow rate in the early diastole (E), maximum flow rate in the systolic phase (A) of the mitral valve.

### Enzyme-linked immunosorbent assay (ELISA)

Two milliliters of blood from the external jugular vein was sampled and immediately centrifuged at 3000 rpm for 10 minutes. The plasma was collected to measure the level of ET-1 and Ang II by an ELISA kit (ADL, American).

To examine the correlations between ET-1 and Ang II in hypoxic plasma, a regression analysis was conducted between the level of ET-1 and Ang II with blood oxygen saturation.

### RT-PCR

Total RNA was extracted from the heart using Trizol reagent (Invitrogen, Carlsbad, CA) and was reverse transcribed by oligo(dT) priming, and first-strand cDNA was synthesized (Applied Biosystems, Foster City, CA). The resultant cDNA was amplified using Taq DNA polymerase, and the fluorescent signal generated was analyzed automatically by ABI 7500 v 2.0.1 software. Relative quantification was used to evaluate the gene expression of the samples.

Primer sequence: *ET-1*, Forward: 5′-GACCACAATGACTTTGGCGTAT-3′;

Reverse: 5′-CGATGGCTTCAGGGATGG-3′;

*GAPDH*, Forward: 5′-ACCTGACCTGCCGTCTAGAA-3′;

Reverse: 5′-TCCACCACCCTGTTGCTGTA-3′

### Statistical analysis

SPSS 22.0 software (SPSS, Chicago, USA) was used for data analysis. Different parameters were determined for normally distributed data. All data were expressed as the mean ± SD. ANOVA was performed to compare groups after normality and variance equality were tested. Correlation was analyzed between blood oxygen saturation and the level of ET-1 and Ang II in the heart. A p value < 0.05 was considered statistically significant.

### Ethical approval

All applicable international, national, and/or institutional guidelines for the care and use of animals were followed. This article does not contain any studies with human participants performed by any of the authors.

## Results

### Induced OSAHS-like symptoms were alleviated by MADs

In Group OSAHS, snoring and apnea progressed over time during sleep, and they showed interrupted sleep. There were no OSAHS-like symptoms in the control animals or in Group MAD. This finding suggested that OSAHS was successfully induced by palatal gel injection and that MADs were effective in alleviating the OSAHS-like symptoms in this study.

**Figure 1:**
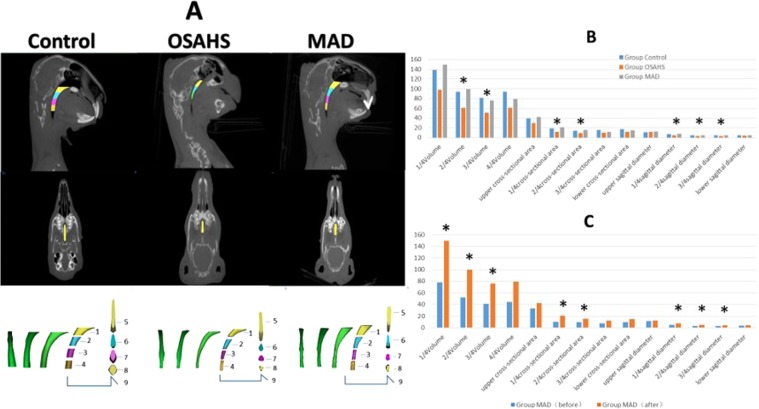
CBCT of upper airway. (**A**) Representative images of upper airway CBCT in three groups (control group, Group OSAHS, and Group MAD). The images of upper airway 3D reconstruction are presented as follows: 1. 1/4 volume; 2. 2/4 volume; 3. 3/4 volume; 4. 4/4 volume; 5. upper cross-sectional area, upper transverse diameter and upper sagittal diameter; 6. 1/4 cross-sectional area, 1/4 transverse diameter and 1/4 sagittal diameter; 7. 2/4 cross-sectional area, 2/4 transverse diameter and 2/4 sagittal diameter; 8. 3/4 cross-sectional area, 3/4 transverse diameter and 3/4 sagittal diameter; 9. lower cross-sectional area, lower transverse diameter and lower sagittal diameter. (**B**) The upper airway volume at 2/4 and 3/4 significantly decreased in Group OSAHS. The sagittal diameter of the upper airway at 1/4, 2/4 and 3/4 significantly decreased in Group OSAHS. There was no difference between Group MAD and the control group. *p < 0.05. (**C**) The upper airway volume and sagittal diameter at 1/4, 2/4 and 3/4 significantly increased, and the cross-sectional area at 1/4 and 2/4 significantly decreased after MAD treatment in Group MAD. *p < 0.05.

**Figure 2:**
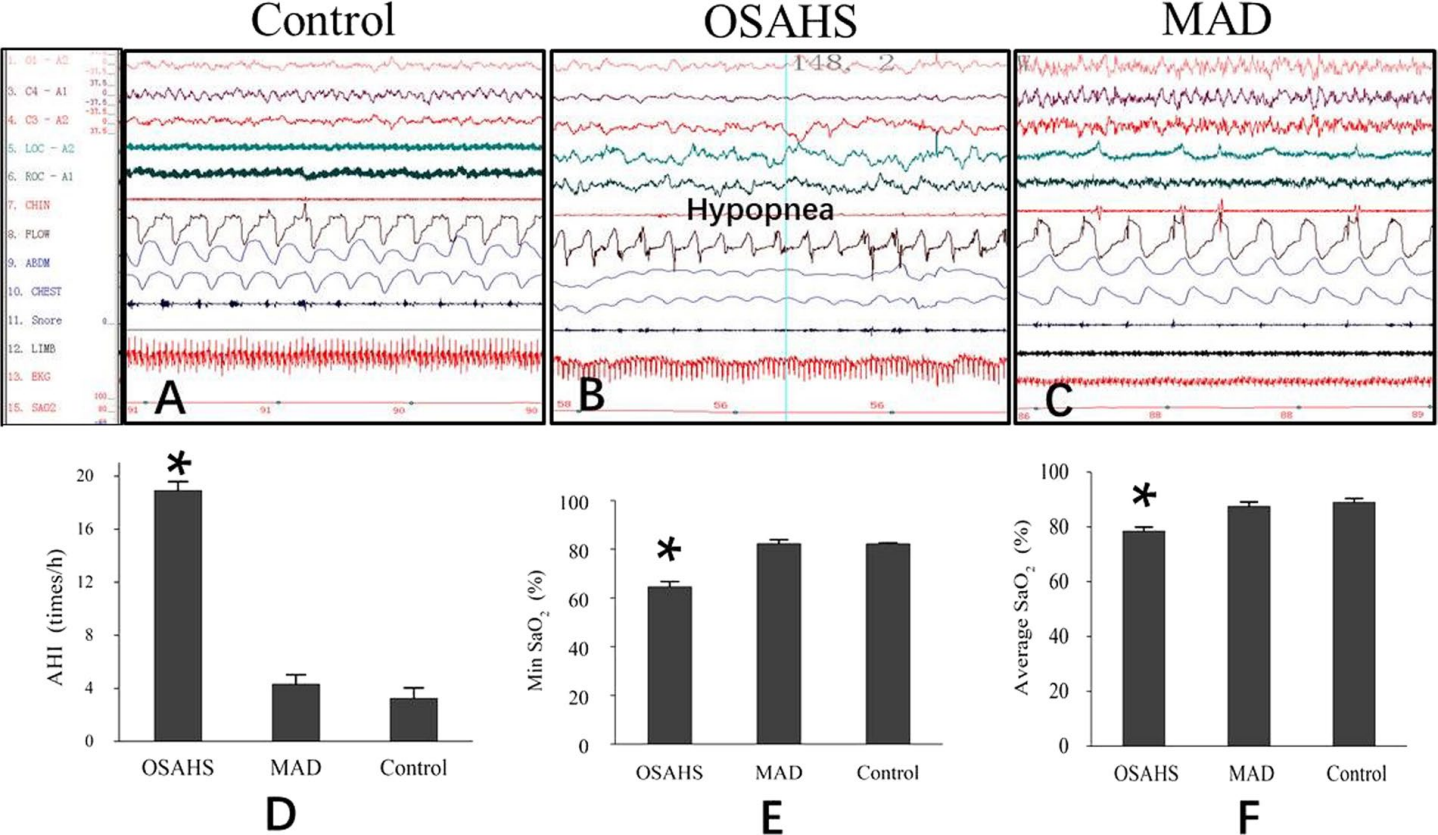
Polysomnography of three groups. (**A**) Representative pattern of 30 s recording in the control group. (**B**) Representative pattern of 30 s recording in Group OSAHS. There was a decrease in nasal airflow (hypopnea) with an associated increase in respiratory effort. (**C**) Representative pattern of 30 s recording in Group MAD. Breathing and air flow are even and smooth in the control group and Group MAD. LOC and ROC: left and right electro-oculogram. C3/A2, C4/A1, O1/A2: electroencephalogram leads. FLOW: nasal air flow. CHEST and ABDM: chest and abdominal strain gauges. Snore: microphone recordings of snoring. (**D**) Significant increases in AHI were seen in Group OSAHS compared with the control group (P < 0.05), and the increased Min SaO_2_ (**E**) and Average SaO_2_ (**F**) in Group OSAHS were corrected significantly by MAD treatment. All of these results showed that MADs significantly improved respiratory function and sleep parameters. *p < 0.05 vs control group.

Similar to our previous studies, the upper airway space of experimental animals was significantly decreased in Group OSAHS (*p* < 0.05) (Fig. 1). The retropalatal space of Group MAD was enlarged by advancing the mandible, and there was no significant difference between Group MAD and the control animals (*p* > 0.05). It was also shown that there was a significant increase in AHI and a decrease in SaO_2_ in Group OSAHS (*p* < 0.05) by PSG, but there were no significant differences in AHI and SaO_2_ between Group MAD and the control group (*p* > 0.05) (Fig. 2). Accordingly, nasal airflow decreased when apnea or hypoxia appeared, with an associated increase in respiratory effort in Group OSAHS. These results suggest that the OSAHS model was successfully induced and that MAD fabrication was feasible and effective.

### OSAHS-induced myocardial hypoxic injury can be prevented by MAD treatment

**Figure 3:**
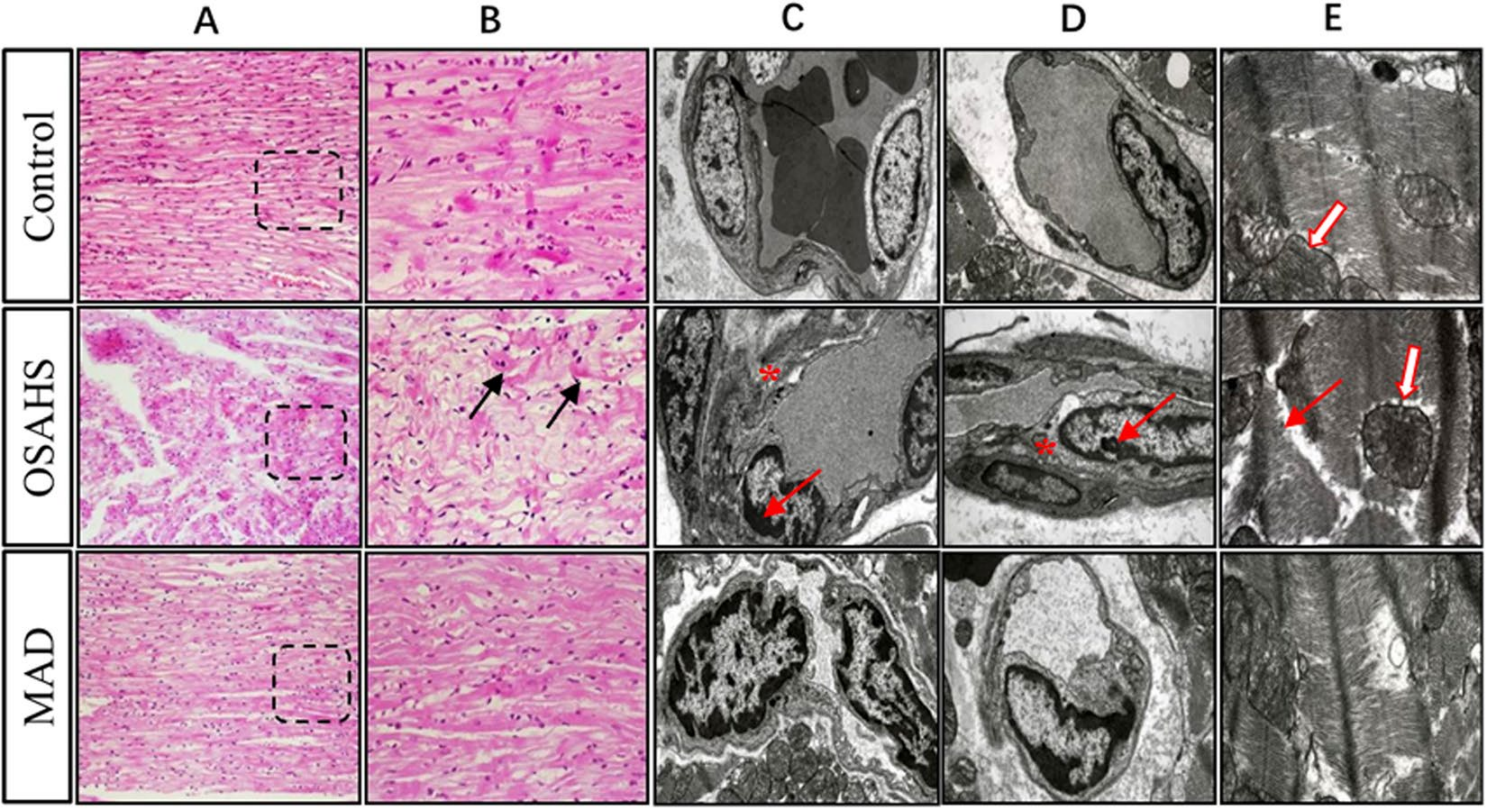
Histopathological changes in the cardiovascular system in the three groups. Hematoxylin-eosin staining by light microscopy showed that cardiac muscle fibers were integrated and arranged in an orderly manner in the control group (**A**,**B**). In Group OSAHS, massive collapsed and fissured cardiac muscle fibers were detected. (**A**,**B**) The arrangement of cardiac muscle fibers was somewhat disordered, and few muscle fibers were collapsed and fissured in Group MAD. (**A**,**B**) The ultrastructure of the capillary among the myocardial fibers in transmission electron microscopy showed the significant thickness of the basement membrane (* shown in **C**,**D**), the pyknosis of nuclear chromatin and the margination phenomenon under the nuclear membrane (arrow shown in **C,D**) in Group OSAHS; The pyknosis of nuclear chromatin and the margination phenomenon under the nuclear membrane and the slight thickness of the basement membrane in Group MAD. (**C**,**D**) The ultrastructure of myocardial fibers showed that the myofibrils were arranged in an orderly manner in the control group. Dissolved and destroyed myofibrils were in disorder in Group OSAHS (arrow shown in **E**), and the fusion and disappearance of the cristae along with the membrane of mitochondria are shown (hollow arrow shown in **E**). These results suggest that myocardial injury associated with OSAHS can be rescued by MADs.

To explore the pathophysiological damage to the heart caused by OSAHS, histological examinations were performed (Fig. 3). In the control group, myocardial fibers were intact and well arranged. In Group OSAHS, the structure of the myocardium was destroyed, such as massive collapsed, fissured and disordered cardiac muscle fibers. However, these changes were not detected in the myocardium in Group MAD. These results suggest that myocardial injury associated with OSAHS can be prevented by MADs.

The ultrastructure of the capillary among the myocardial fibers showed that the pinocytotic vesicles, mitochondria and rough surfaced endoplasmic reticulum were intact in the cytoplasm of endothelial cells in the control group (Fig. 3). In Group OSAHS, the following changes could be observed: local swelling in the cytoplasm of endothelial cells, the fusion and disappearance of the cristae along with the membrane of mitochondria, the pyknosis of nuclear chromatin and the margination phenomenon under the nuclear membrane, as well as the thickening of the capillary basement membrane. In Group MAD, the basement membrane thickened slightly, suggesting that the ultrastructure of the capillary among the myocardial fibers improved with MAD therapy.

The ultrastructure of myocardial fibers showed that the myofibers were well arranged and intercalated disks were clear in the control group. In Group OSAHS, the myofibril arrangement was disorderly, and the structure was blurred. Many myofibrils were dissolved, destroyed and had vanished. Some densified mitochondria and the fusion and disappearance of the cristae along with the membrane of mitochondria were present in the myocardial fibers in Group OSAHS. The myofibrillar arrangement was slightly disordered in Group MAD. A small amount of myocardial fibers collapsed and disappeared. Part of the myomere was unclear and unrecognizable. Local fusion and disappearance of the cristae along with the membrane of mitochondria were detected.

### OSAHS-induced heart dysfunction was prevented by MAD treatment

**Figure 4:**
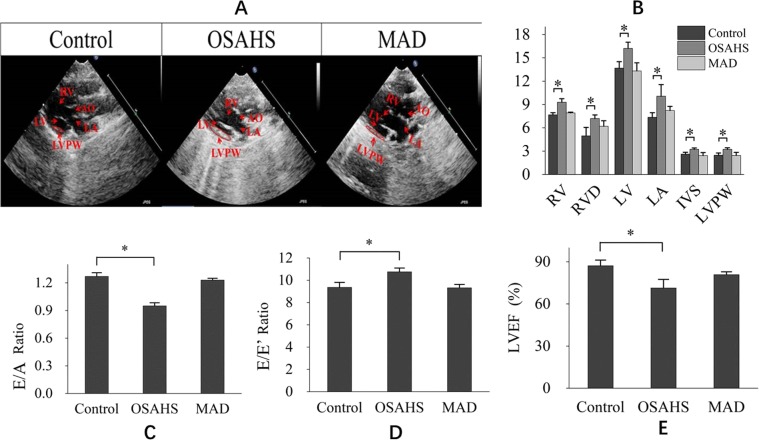
Changes in cardiovascular structure and function in three groups. (**A**) Representative echocardiography images of the heart in the three groups. (**B**) The structure of the heart in the three groups. RV, right ventricle; RVD, right ventricle diameter; LV, left ventricle; LA, left atrium; IVS, thickness of the interventricular septum. The right ventricle diameter increased, and the thickness of the left and right ventricular wall increased, which showed that there was left ventricular and right ventricular hypertrophy in Group OSAHS, and there was no significant difference in the right ventricle diameter and the thickness of the ventricular wall between Group MAD and the control group. (**C**) Peak *E*: the peak of early diastolic velocities; Peak *A*: the peak of late diastolic velocities; *a*’: late diastolic mitral annulus velocities; E/*a*’ ratio: an estimate of left ventricular diastolic function; (**D**) *e*’: early diastolic mitral annulus velocities; E/*e*’ ratio: an estimate of LV end-diastolic pressures. (**C,D**) Showed that the diastolic function of the left ventricle degraded; however, there was no significant difference between Group MAD and the control group. (**E**) LV systolic performance was assessed by the left ventricular ejection fraction (LVEF). (**E**) showed that the diastolic function of the left ventricle decreased, which was not found in Group MAD and the control group.

Myocardial changes in OSAHS animals reflected their heart dysfunctions. In this study, echocardiographic images were recorded to determine ventricular wall thickness and left ventricular function (Fig. 4). A combined measure of left ventricular systolic and diastolic function performance was performed in this study. The right ventricle enlarged, and the ventricular wall thickness increased, suggesting ventricular hypertrophy in Group OSAHS. There was no significant difference in right ventricle diameter or ventricular wall thickness between Group MAD and the control group. The E/*a*’ ratio was an estimate of left ventricular diastolic function, and the E/*e*’ ratio was an estimate of LV end-diastolic pressures. A decreased E/*a*’ ratio and increased E/*e*’ ratio were found in the OSAHS group, which suggested that the diastolic function of the left ventricle had degraded. However, there was no significant difference between Group MAD and the control group. LV systolic performance was assessed by the left ventricular ejection fraction (LVEF). Decreased diastolic function of the left ventricle was found in Group MAD and the control group. Left ventricular systolic performance was reduced in Group OSAHS, and there was no significant difference between Group MAD and the control group (Fig. 4).

### Increased systemic cytokines were related to OSAHS and could be downregulated by MAD treatment

**Figure 5:**
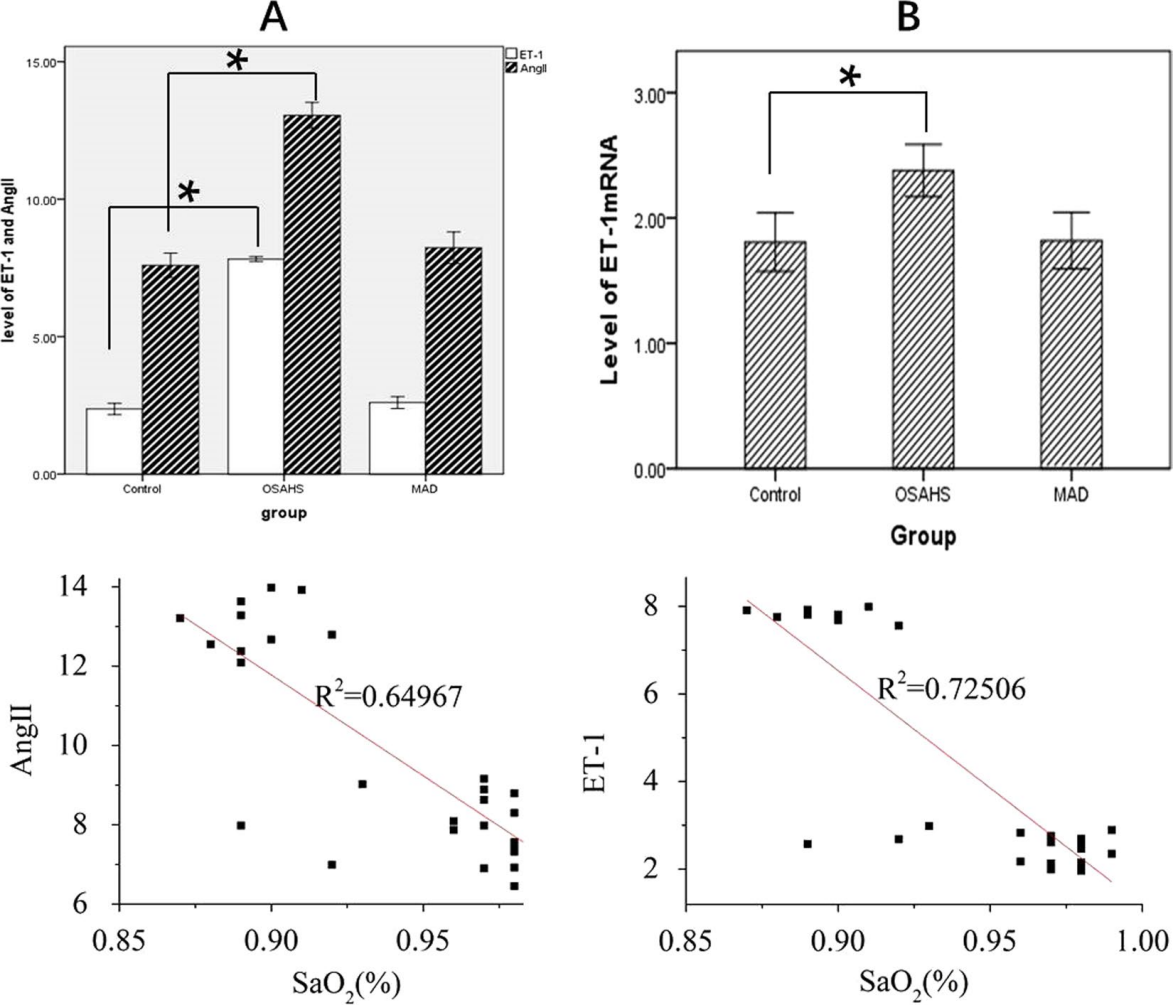
Systemic cytokines in three groups. The levels of ET-1 and Ang II in plasma (**A**) were detected by ELISA. The level of ET-1 mRNA expression (**B**) was detected by RT-PCR. *p < 0.05. (**C**) There was a negative correlation between the average blood oxygen saturation and the level of Ang II in plasma. (**D**) There was a negative correlation between the average blood oxygen saturation and the level of ET-1 in plasma.

Chronic intermittent hypoxia (CIH) could induce sympathetic nervous system overactivity, which resulted in the overproduction of angiotensin II (Ang II) and endothelin-1 (ET-1)^29^. Endothelin-1 (ET-1), a potent vasoconstrictor secreted from the endothelium, plays an important role in the pathogenesis of cardiovascular alterations^30^. Ang II is a multifunctional peptide that regulates vascular tone and blood pressure. Therefore, we speculated that OSAHS-induced myocardial injuries were associated with the overproduction of these systemic cytokines due to hypoxia. In this study, we first used ELISA and RT-PCR to examine the changes in the circulating levels of ET-1 and Ang II to explore their association with OSAHS-induced cardiovascular injury. In Group OSAHS, there was a significant increase in ET-1 and Ang II in plasma compared to the control group, while there was no significant difference between Group MAD and the control group. The level of ET-1 mRNA expression was higher in Group OSAHS than in Group MAD and the control group (Fig. 5). Second, to analyze the relationship between these systemic cytokines and hypoxia, we examined the correlation of plasma levels of ET-1 and Ang II with blood oxygen saturation. We found that blood oxygen saturation was negatively correlated with the level of ET-1 and Ang II in plasma (Fig. 5). This suggested that hypoxia in OSAHS was associated with elevated systemic cytokines, which might cause the OSAHS-induced myocardium damage in this study.

## Discussion

OSAHS is characterized by chronic intermittent hypoxia, sleep disruption and increased-effort breathing. The CIH model was most commonly used to investigate cardiovascular diseases caused by OSAHS^31,32^, although it could not completely simulate all symptoms in OSAHS patients. Due to the limitations of clinical studies, OSAHS animal models provide useful tools to study the consequences of OSAHS and their underlying pathophysiological mechanisms. In addition, animal models could mimic some manifestations of OSAHS in humans under well-controlled experimental conditions. Therefore, in the present study, we used the OSAHS model developed in our previous study^26^. This model mimicked the characteristics of OSAHS to a large extent, including chronic intermittent hypoxia, sleep disruption, and increased-effort breathing.

Consistent with our previous studies, CBCT showed that the upper airway space was significantly decreased in Group OSAHS. This result suggested that gel injection induced upper airway stenosis successfully. The MADs protruded the mandible and enlarged the space of the retropalatal upper airway. PSG showed a significant increase in AHI and a decrease in SaO_2_ by reduced nasal airflow, increased respiratory effort and sleep disruption in Group OSAHS. These findings were in accordance with the clinical features observed in OSAHS patients. However, all the OSAHS-like symptoms were alleviated after they were given MAD treatment, which suggested that the OSAHS model and MAD treatment were effective. In this study, we took advantage of the OSAHS animal model to examine the effects of OSAHS on the myocardium. It is well known that CPAP treatment of OSAHS patients has an impact on cardiovascular or cerebrovascular diseases such as hypertension^33^, coronary artery disease^34^, or cerebral infarct^35^. Although there was no definitive evidence to determine whether OSAHS directly caused cardiovascular disease, the data from this study implied that OSAHS could result in abnormal myocardium structure. This histological cardiac structure disorder and left ventricular hypotrophy in OSAHS rabbits were not detected in Group MAD, indicating that early treatment of MAD can prevent OSAHS-induced cardiovascular complications.

In this study, echocardiographic images were recorded to determine the relative ventricular wall thickness. LV systolic performance was assessed by LVEF, and LV diastolic function was evaluated by E/*a*’ and E/*e*’. It might be more convincing to evaluate overall cardiac dysfunction by a combined measure of left ventricular systolic and diastolic function in this study, rather than systolic or diastolic measures alone. Left ventricular systolic and diastolic dysfunction were found in OSAHS rabbits, and MAD treatment could rescue left ventricular systolic and diastolic function. The causes of left ventricular dysfunction included intermittent hypoxemia due to apnea or hypopnea, changes in left ventricular afterload and sleep fragmentation at night. Studies have shown that LV diastolic dysfunction and LV hypertrophy were detected in patients with OSAHS^36^. Furthermore, significantly elevated left ventricular end-systolic volume (LVESV) along with decreased LVEF were detected in a rat chronic intermittent hypopnea model^37^. However, there was no significant difference in LV function, although LV hypertrophy was found^37,38^. In addition, another study showed the beneficial effects of CPAP on the cardiac functions of patients with OSAHS^39^.

The possible mechanisms of OSAHS-induced deleterious effects on the cardiovascular system may involve two major components. First, chronic intermittent hypoxia during repeated apnea or hypopnea resulted in sympathetic overdrive and endothelial cell dysfunction. Second, large negative intrathoracic pressures were generated during inspiratory efforts when apneas appeared, which increased transmural pressures across the myocardium and increased afterload, thus having a negative effect on myocardial structure and function. Additionally, frequent arousals from sleep led to increased sympathetic activity. LV systolic and diastolic function were significantly improved by MAD therapy. The beneficial effects of MADs may result from the following: the reopening of the constricted upper airway of OSAHS, consequently improving blood oxygen saturation, and the avoidance of the elevation of intrathoracic pressure, left ventricular afterload and sympathetic nerve activity. All of these factors could prevent the deleterious effects of OSAHS on cardiac structure and function.

Sympathetic hyperactivity, oxidative stress and systemic inflammation have been regarded as potential mechanisms of hypertension and other cardiovascular diseases^39^. Sympathetic nervous system overactivity could result in the overproduction of Ang II and ET-1, which are important to vascular tone and blood pressure. CIH-induced cardiac dysfunction might be associated with decreased coronary tone. An intact coronary endothelium was shown to be important in regulating coronary blood flow during OSAHS^8^. Indeed, the level of ET-I and Ang II increased significantly in Group OSAHS compared with the control group and Group MAD, and there was a negative correlation of ET-1 and Ang II with blood oxygen saturation in the current study. This suggested that myocardium damage was probably due to the increased levels of ET-1 and Ang II in plasma, which were associated with intermittent hypoxia caused by OSAHS. First, intermittent hypoxia leads to damage to endothelial cells and functional disorders such that endothelial cells release too much ET-1. Second, hypoxia can increase the genetic transcription of ET-1 in endothelial cells^40^. Third, intermittent hypoxia may indirectly affect the synthesis^41^ and the secretion of ET-1 by affecting adrenaline and noradrenaline. Excess ET-1 binding specifically to ET receptor-A could stimulate local blood vessel contraction and extracellular calcium influx, which eventually resulted in the overload of intracellular calcium and damage to myocardial cells^30^. Ang II is mainly produced by endothelial cells and is the central part of the renin-angiotensin-aldosterone system (RAAS). It was reported that the levels of plasma renin activity (RA) and Ang II increased significantly in OSAHS patients and could be reduced through CPAP treatment, suggesting that OSAHS may increase RAAS activity^29^. On the other hand, myocardial damage could further exacerbate the hypoxia-increasing ET-I and Ang II levels in the plasma, and thus, a vicious circle is formed. Our findings suggested that early treatment of MADs could prevent serious cardiovascular complications caused by OSAHS.

Although CPAP is widely used to treat OSAHS and shows higher efficacy than MAD in treating OSAHS, it has some shortcomings. First, its efficacy is compromised by poor patient tolerance and compliance^25^. Second, it is hard to be test in animal study. In addition, CPAP can modify cardiac transmural pressure and therefore may directly influence cardiac functions^42^. Therefore, to test better tolerated MAD treatment is more practical. In this and our previous investigations^26^, we provided evidences that MAD normalized AHI and SaO2 status of OSAHS and did not change cardiac transmural pressure, it suggests that MAD remediated the adverse respiratory effects of OSAHS but not affecting the cardiac function in current model.

In addition, we found that the cardiac dysfunctions and their alleviation discovered in this model is caused by respiratory airway relief, as we used Et1 and Ang II, which are correlated with average blood oxygen saturation, to evaluate their elevation in OSAHS and the reversing by MAD treatment. The results suggested that OSAHS-induced cardiac complications prevention is related to respiratory events prevention, and this is probably independent from the type of preventive upper airway obstruction mechanical treatment. Therefore, MAD can be recommended for severe OSAS treatment if supported with more clinical studies.

## Data Availability

All data generated or analyzed during this study are included in this published article.
